# Gratuitous Dentistry in the French Schools

**Published:** 1882-02

**Authors:** 


*Gratuitous Dentistry in the French Primary Schools.*—We are in receipt of a copy of a petition from M. E. Tallebois, a Parisian Dentist, to the Municipal Council of Paris, advocating the establishment of such gratuitous services, an offer which is very commendable on the part of the proposed—especially when we learn that Mr. Taillebois offers to defray the expenses from his own resources.

